# Sleep patterns and risk of chronic disease as measured by long-term monitoring with commercial wearable devices in the All of Us Research Program

**DOI:** 10.1038/s41591-024-03155-8

**Published:** 2024-07-19

**Authors:** Neil S. Zheng, Jeffrey Annis, Hiral Master, Lide Han, Karla Gleichauf, Jack H. Ching, Melody Nasser, Peyton Coleman, Stacy Desine, Douglas M. Ruderfer, John Hernandez, Logan D. Schneider, Evan L. Brittain

**Affiliations:** 1 Yale School of Medicine, Yale University, New Haven, CT USA; 2 Brigham and Women’s Hospital, Boston, MA USA; 3 Vanderbilt Institute for Clinical and Translational Research, Vanderbilt University Medical Center, Nashville, TN USA; 4 Center for Digital Genomic Medicine, Department of Medicine, Vanderbilt University Medical Center, Nashville, TN USA; 5 Division of Cardiovascular Medicine, Vanderbilt University Medical Center, Nashville, TN USA; 6 Division of Genetic Medicine, Department of Medicine, Vanderbilt Genetics Institute, Vanderbilt University Medical Center, Nashville, TN USA; 7 Google, Mountain View, CA USA; 8 Nelson Connects, San Francisco, CA USA; 9 Department of Psychiatry and Behavioral Sciences, Vanderbilt University Medical Center, Nashville, TN USA; 10 Department of Biomedical Informatics, Vanderbilt University Medical Center, Nashville, TN USA; 11 Sleep Medicine Center, Department of Psychiatry and Behavioral Sciences, Stanford University School of Medicine, Redwood City, CA USA

**Keywords:** Epidemiology, Lifestyle modification

## Abstract

Poor sleep health is associated with increased all-cause mortality and incidence of many chronic conditions. Previous studies have relied on cross-sectional and self-reported survey data or polysomnograms, which have limitations with respect to data granularity, sample size and longitudinal information. Here, using objectively measured, longitudinal sleep data from commercial wearable devices linked to electronic health record data from the All of Us Research Program, we show that sleep patterns, including sleep stages, duration and regularity, are associated with chronic disease incidence. Of the 6,785 participants included in this study, 71% were female, 84% self-identified as white and 71% had a college degree; the median age was 50.2 years (interquartile range = 35.7, 61.5) and the median sleep monitoring period was 4.5 years (2.5, 6.5). We found that rapid eye movement sleep and deep sleep were inversely associated with the odds of incident atrial fibrillation and that increased sleep irregularity was associated with increased odds of incident obesity, hyperlipidemia, hypertension, major depressive disorder and generalized anxiety disorder. Moreover, J-shaped associations were observed between average daily sleep duration and hypertension, major depressive disorder and generalized anxiety disorder. These findings show that sleep stages, duration and regularity are all important factors associated with chronic disease development and may inform evidence-based recommendations on healthy sleeping habits.

## Abstract (web summary)

In a cohort of 6,785 participants from the All of Us Research Program whose sleep was monitored by a Fitbit over a median of 4.5 years, sleep duration, stages and irregularity were associated with the incidence of obesity and a number of cardiovascular and psychological disorders.

## Main

Sleep health has been associated with all-cause mortality and chronic diseases, including psychiatric and cardiometabolic disorders^1–8^. Many previous studies have focused on sleep durations and have reported J-shaped associations, in which individuals with shorter (≤6 h) or longer (≥9 h) average daily sleep duration are at higher risk for a variety of poor health outcomes^1,2,6^. These studies have helped inform recommendations suggesting that approximately 7–9 h of daily sleep is appropriate for most adults^9^.

Less is known about the associations between chronic disease and other real-world sleep patterns, including sleep regularity and stages (for example, N1, N2, N3, rapid eye movement (REM)). Most epidemiologic studies on sleep and chronic diseases have relied on self-reported sleep data^1–7^. For example, several landmark epidemiologic studies, including the Nurses’ Health Study and the National Health and Nutrition Examination Survey, simply asked participants to estimate their typical sleep duration over a 24-h period^1,4^. Self-reported sleep data are unable to capture sleep stages and offer inaccurate representations of longitudinal sleep patterns. Studies using polysomnography rely on a limited number of nights of sleep data because of time and monetary constraints^10,11^. Other studies have used actigraphy-based sleep measures, but these studies rely on sleep data from 7 to 14 days of actigraphy data, which cannot capture longitudinal sleep patterns including seasonal or individual variations in sleep^8,12^. Newer ambulatory electroencephalographic devices have comparable performance to gold-standard polysomnograms^13^, but there have been no large-scale studies using these devices to date because of their limited use by the general population. Recent developments in commercial wearable devices, such as Fitbit, now enable objective longitudinal measurements of sleep patterns in the general population with good performance when compared with polysomnograms^14–21^. The growing popularity of these devices now makes possible large-scale epidemiological studies on associations between wearables-derived metrics and chronic diseases^22–25^.

The All of Us Research Program (AoU) is a National Institutes of Health-funded initiative to gather health data from more than one million diverse persons living in the United States^26,27^. Participants are enrolled digitally and invited to share multiple longitudinal sources of health-related information, including electronic health records (EHRs), genomics, physical measures and participant surveys^26^. Participants who owned Fitbit devices were invited to voluntarily share their Fitbit data, including physical activity and sleep patterns with a median monitoring time of 4.5 years. By connecting Fitbit data with EHRs, the AoU has made it possible, for the first time, to perform large-scale, longitudinal studies of objectively measured physical activity and sleep patterns in association with clinical outcomes data^22^.

In this study, we leveraged the longitudinal EHR data and daily sleep patterns in the AoU to investigate associations between sleep patterns over time and incident chronic disease. Our approach included both phenome-wide association studies (PheWAS) aimed at discovery and targeted assessment of specific chronic diseases associated with poor sleep with findings relevant to the general US population.

## Results

**Fig. 1:**
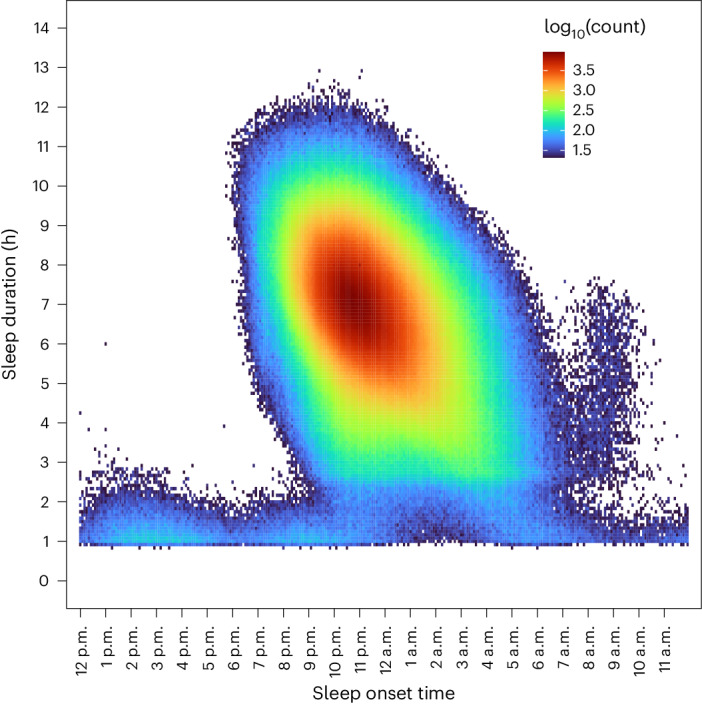
Heatmap of sleep onset time and total sleep duration for all person-nights. Heatmap of sleep duration is plotted versus sleep onset time across all person-nights. All sleep periods were flagged as ‘main sleep’ by Fitbit devices.

**Extended Data Fig. 1:**
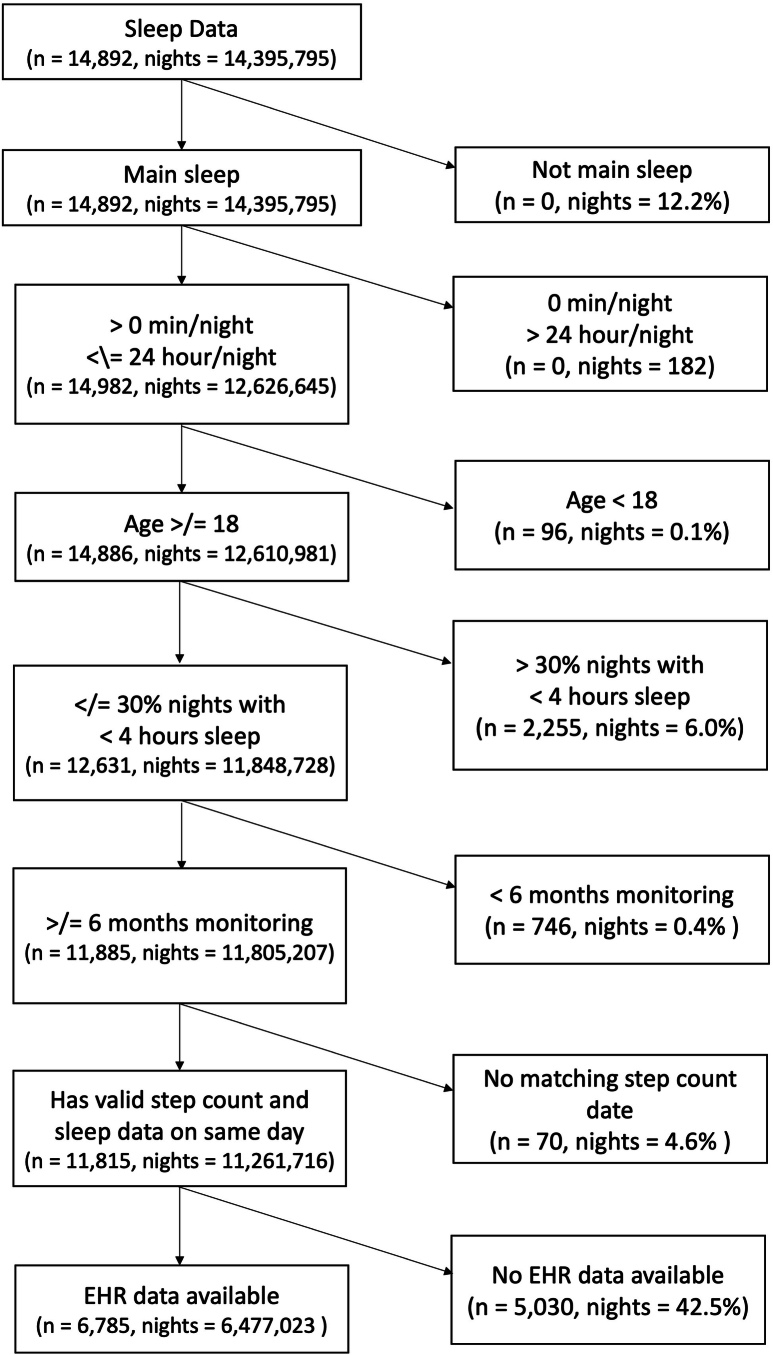
CONSORT diagram showing inclusion/exclusion criteria. The figure shows the inclusion and exclusion criteria for participants and participants-nights for the study. Boxes on the left-hand side of the figure describe the inclusion criteria and boxes on the right-hand side of the figure describe the exclusion criteria.

**Extended Data Fig. 2:**
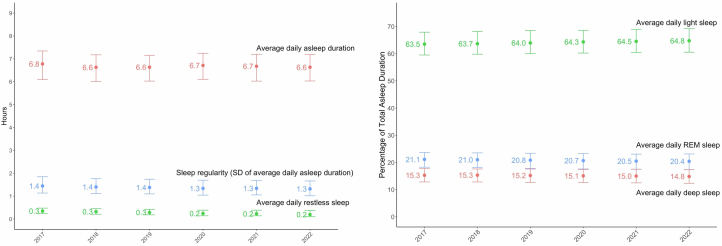
Population medians of Fitbit-derived sleep metrics from 2017 to 2022. Collection for Fitbit data for sleep duration, restless sleep duration, and sleep irregularity started prior to 2017 whereas collection for Fitbit-derived sleep stages started in 2017. Point and number indicate the population median. Errors bars indicate the 25th and 75th percentile. Sample sizes were 3,617 for 2017, 4,345 for 2018, 5,319 for 2019, 5,647 for 2020, 5,585 for 2021, and 4,626 for 2022.

We identified 14,892 individuals with Fitbit-derived sleep data. A total of 6,785 adult participants with linked EHR data, a Fitbit monitoring period of at least 6 months and fewer than 30% of nights with <4 h of sleep were included for analysis, resulting in 6,477,023 person-nights (Extended Data Fig. 1). The median age was 50.2 years (interquartile range = 35.7, 61.5) (Table 1). Most participants were female (71%), white (84%) and college educated (71%). The median Fitbit monitoring length was 4.49 (2.53, 6.45) years and median average daily step count was 7,798 (5,898, 9,947) steps per day. Median sleep patterns were 11:10 p.m. (10:30, 00:00) for sleep onset time (Fig. 1), 6.7 (6.2, 7.2) h for sleep duration, 0.3 (0.2, 0.5) h for restless sleep duration and 1.5 (1.2, 1.8) for sleep irregularity (standard deviation (s.d.) of average daily sleep duration in hours). The median proportion of weekdays with sleep onset during ‘traditional’ hours (8:00 p.m. to 2:00 a.m.) was 93.5% (85.2, 97.3). For sleep stages, the median percentage spent in REM, light and deep sleep were 20.7% (17.8, 23.1), 64.2% (60.3, 68.5) and 15.1% (12.7, 17.5), respectively. Population median for Fitbit-derived sleep metrics remained relatively stable from 2017 to 2022 (Extended Data Fig. 2). There were significant differences in median sleep duration when stratified by participant demographics (self-reported sex, self-reported race/ethnicity, education) and lifestyle factors (smoking, alcohol intake) (Table 1).

**Table 1:** Baseline characteristics of study participants

Variable^a^	Fitbit sleep participants	Sleep duration
Subjects	6,785	
Age	50.2 (35.7, 61.5)	
Sex		
Female	4,802 (70.8)	6.76 (6.23, 7.22)*
Male	1,801 (26.5)	6.51 (5.98, 7.01)
Unknown	182 (2.7)	
Race		
White	5,673 (83.6)	6.75 (6.23, 7.22)*
Black or African American	296 (4.4)	6.06 (5.67, 6.55)
Other	563 (8.3)	6.52 (6.02, 7.02)
Unknown	253 (3.7)	
Education		
College degree	4,832 (71.2)	6.71 (6.19, 7.19)*
Some college	344 (5.1)	6.65 (6.08, 7.16)
No college	1,420 (20.9)	6.62 (6.06, 7.13)
Unknown	189 (2.8)	
Smoking		
Never	4,536 (66.5)	6.72 (6.18, 7.19)*
Ever (≥100 cigarettes)	2,132 (31.7)	6.63 (6.07, 7.13)
Unknown	117 (1.7)	
Alcohol (How often do you have a drink containing alcohol?)		
Never	704 (10.4)	6.53 (6.00, 7.13)*
Monthly or less	1,962 (28.9)	6.63 (6.07, 7.15)
2–4 times per month	1,724 (25.4)	6.74 (6.23, 7.17)
2–3 times per week	1,188 (17.5)	6.75 (6.23, 7.23)
≥4 times per week	989 (14.6)	6.78 (6.27, 7.23)
Unknown	218 (3.2)	
Fitbit sleep monitoring length (years)	4.49 (2.53, 6.45)	
Daily steps	7,798 (5,898, 9,947)	
Average daily sleep pattern (h)		
Restless	0.3 (0.2, 0.5)	
Sleep	6.7 (6.2, 7.2)	
Average s.d. of daily sleep duration (h)	1.5 (1.2, 1.8)	
Average daily sleep stage (%)		
REM	20.7 (17.8, 23.1)	
Light	64.2 (60.3, 68.5)	
Deep	15.2 (12.7, 17.5)	

^a^All values are reported as median (interquartile range) or *N* (%) unless otherwise specified. Percentages may not add to 100% because patients can decline to answer survey questions.

^*^Significant difference (*P* < 0.05) in median sleep duration between categories from Mann–Whitney *U-*tests for variables ≤2 categories and Kruskal–Wallis test for variables with >2 categories.

### Association of sleep patterns with incident disease

**Fig. 2:**
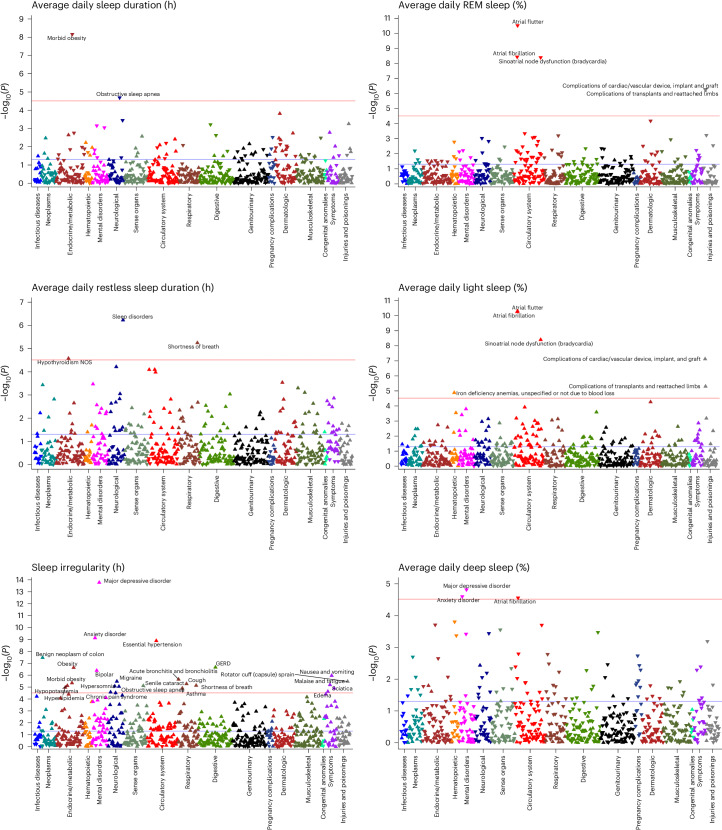
Phenome-wide analyses to explore relation between Fitbit-derived sleep metrics and incident disease. Phenome-wide analyses were performed to identify associations between each Fitbit-derived sleep metric and incident disease. All phenome-wide analyses were performed using multiple logistic regression models adjusted for age, sex, average daily step count across the entire monitoring period and EHR length. All reported *P* values were based on two-tailed probability. The Bonferroni significance line of 3.06 × 10^−^^5^ is indicated by a red line and a *P* value of 0.05 is indicated by the blue line. Upwards pointing triangles indicate OR >1 and downwards pointing triangles indicated OR <1. GERD, gastroesophageal reflux disease; NOS, not otherwise specified.

**Fig. 3:**
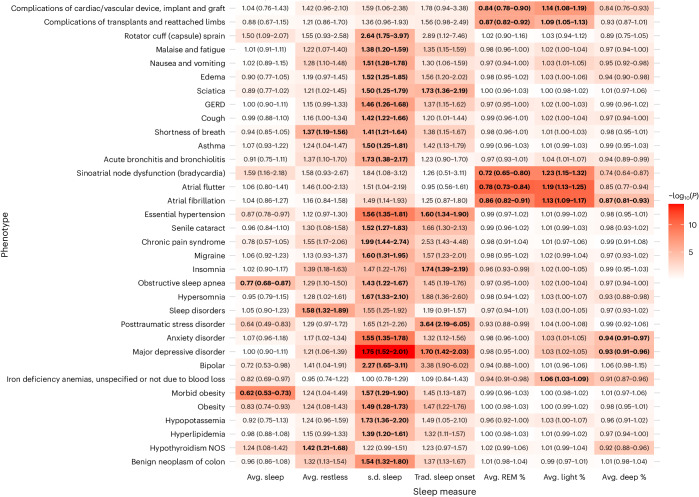
Heatmap of significant incident phenotypes across all Fitbit-derived sleep metrics. Heatmap of −log_10_(*P*) is overlaid on a table of significant associations between all incident phenotypes and Fitbit-derived sleep metrics. OR values (95% CI) are reported within each heatmap table box. OR values for average (avg.) sleep, average restless and s.d. of sleep duration are reported per hour change. OR values for average percent sleep stage are reported per 10% change. All reported ORs are from multiple logistic regression models adjusted for age, sex, average daily step count across the entire monitoring period and EHR length. Boxes that include bold text indicate associations that were significant after Bonferroni correction line of 3.06 × 10^−5^. All reported *P* values were based on two-tailed probability. Trad., traditional.

Discovery-focused PheWAS identified 48 significant associations with Fitbit-derived sleep patterns after Bonferroni correction (Fig. 2), including 2 associations for sleep duration, 3 for restless sleep duration, 14 for sleep stages, 24 for sleep irregularity and 5 for traditional versus nontraditional sleep onset. There were several phenotypes with significant associations across multiple sleep patterns, such as the association between insomnia and average restless sleep duration, sleep irregularity and traditional sleep onset proportion (Fig. 3).

Each hour increase in average daily sleep duration was associated with decreased odds of receiving a new diagnosis for morbid obesity (odds ratio (OR) = 0.62; 95% confidence interval (CI) = 0.53–0.73) and obstructive sleep apnea (0.77; 0.68–0.87). Increased average daily restless sleep (per hour) was associated with increased odds of sleep disorders (1.58; 1.32–1.89), hypothyroidism (1.42; 1.21–1.68) and shortness of breath (1.37; 1.19–1.56).

Increased sleep irregularity (per hour change in s.d. of daily sleep duration) was associated with a variety of incident psychiatric, sleep and metabolic disorders. Chronic conditions associated with increased sleep irregularity included essential hypertension (1.56; 1.35–1.81), hyperlipidemia (1.39; 1.20–1.61) and obesity (1.49; 1.28–1.73). We also observed increased odds of several psychiatric disorders, including major depressive disorder (1.75; 1.52–2.01), anxiety disorder (1.55; 1.35–1.78) and bipolar disorder (2.27; 1.65–3.11). In addition, increased sleep irregularity was associated with conditions that may disrupt sleep, including gastroesophageal reflux disease (1.46; 1.26–1.68), obstructive sleep apnea (1.43; 1.22–1.67), asthma (1.50; 1.25–1.81) and migraine (1.60; 1.31–1.95). The majority (23 of 24) of PheWAS associations for sleep irregularity were still significant after adjustment for average daily sleep duration (Extended Data Table 1).

When examining Fitbit-derived sleep stages, each percent increase in REM sleep was associated with a reduced incidence of heart rhythm and rate abnormalities, including atrial fibrillation (OR 0.86; 95% CI 0.82–0.91), atrial flutter (0.78; 0.73–0.84) and sinoatrial node dysfunction/bradycardia (0.72; 0.65–0.80). Higher light sleep percentage (per percent) was associated with increased odds of atrial fibrillation (1.13; 1.09–1.17), atrial flutter (1.19; 1.13–1.25), sinoatrial node dysfunction/bradycardia (1.23; 1.15–1.32) and iron deficiency anemia (1.06; 1.03–1.09). Higher deep sleep percentage (per percent) was also associated with lower odds of atrial fibrillation (0.87; 0.81–0.93), major depressive disorder (0.93; 0.91–0.96) and anxiety disorder (0.94; 0.91–0.97).

**Extended Data Fig. 3:**
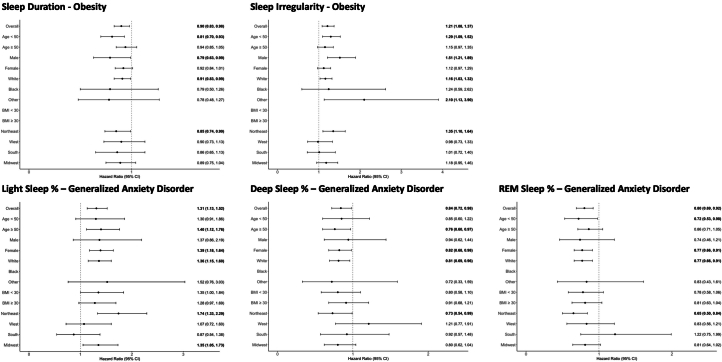
Forest plots from stratified analyses of Cox proportional hazard models for associations between 75th vs. 25th percentile Fitbit-derived sleep metric and chronic disease. The figure shows significant findings from a total of 112 stratified analyses (13 strata 9 significant associations from overall Cox proportional hazard models). The points indicate the hazard ratios and the error bars indicate the 95% confidence intervals. Strata with fewer than 20 cases were excluded to comply with All of Us Data and Statistics dissemination policies. All Cox proportional hazards models were adjusted for age, sex, baseline body mass index (BMI), baseline systolic blood pressure, smoking status, alcohol drinking status, education status, time-varying average daily step count, and prior diagnoses of cancer or coronary artery disease.

When comparing participants with sleep onset proportion greater and less than the median (93.5%), participants who had lower proportions of traditional sleep onset had higher odds for incident major depressive disorder (1.70; 1.42–2.03), post-traumatic stress disorder (3.64; 2.19–6.05), insomnia (1.74; 1.39–2.19), essential hypertension (1.60; 1.34–1.90) and sciatica (1.73; 1.36–2.19) (Extended Data Fig. 3). The association for sciatica was no longer significant after adjusting for average daily sleep duration. Every 10% decrease in the proportion of days with sleep onset within the ‘traditional’ time window was associated with a 19% and 37% increase in odds of hypersomnia (1.19; 1.10–1.27) and circadian rhythm sleep disorders (1.37; 1.20–1.56), respectively.

### Time-varying analysis of sleep patterns and chronic disease

We performed Cox proportional hazard analyses for chronic diseases selected a priori from reported associations in previous studies (Table 2)^1–7,28^. Increased average sleep duration was associated with lower risk for obesity (hazard ratio (HR) = 0.90; 95% CI = 0.83–0.98), whereas increased sleep irregularity was associated with increased risk of obesity (1.21; 1.08–1.37). Increased REM sleep percentage was associated with decreased risk of incident heart failure (HR 0.51; 95% 0.26–0.99) and generalized anxiety disorder (0.80; 0.69–0.92). Increased light sleep percentage was associated with increased risk of incident heart failure (2.30; 1.05–5.04), generalized anxiety disorder (1.31; 1.13–1.52) and atrial fibrillation (1.76; 1.02–3.05). Increased deep sleep percentage was associated with decreased risk of atrial fibrillation (0.59; 0.35–0.99) and generalized anxiety disorder (0.84; 0.72–0.98).

**Table 2:** Cox proportional hazards models for the association between 75th versus 25th percentile Fitbit-derived sleep metric and chronic diseases

	Average sleep duration		Average restless duration		Sleep irregularity (s.d. sleep duration)		Average % REM		Average % light sleep		Average % deep sleep	
Chronic disease	*N*^a^	HR (95% CI)^b^	*N*	HR (95% CI)	*N*	HR (95% CI)	*N*	HR (95% CI)	*N*	HR (95% CI)	*N*	HR (95% CI)
Atrial fibrillation	102/5,513	1.12 (0.87–1.45)	83/6,095	1.13 (0.79–1.60)	102/5,513	1.19 (0.83–1.70)	83/6,095	0.67 (0.43–1.03)	83/6,095	**1.76 (1.02–3.05)**	83/6,095	**0.59 (0.35–0.99)**
Heart failure	68/5,590	0.93 (0.69–1.25)	54/6,182	1.15 (0.74–1.77)	68/5,590	1.12 (0.72–1.73)	54/6,182	**0.51 (0.26–0.99)**	54/6,182	**2.30 (1.05–5.04)**	54/6,182	0.59 (0.30–1.16)
Coronary artery disease	113/5,421	0.96 (0.75–1.23)	90/6,007	1.12 (0.79–1.57)	113/5,421	1.32 (0.94–1.87)	90/6,007	0.91 (0.64–1.30)	90/6,007	1.16 (0.77–1.77)	90/6,007	1.12 (0.76–1.63)
Hypertension	1,049/2,827	1.00 (0.92–1.09)	773/3,137	1.01 (0.90–1.14)	1,049/2,827	1.11 (0.99–1.24)	773/3,137	0.97 (0.87–1.08)	773/3,137	1.06 (0.94–1.20)	773/3,137	0.94 (0.84–1.07)
Obesity	1,176/4,270	**0.90** (**0.83–0.98)**	859/4,748	1.13 (1.00–1.27)	1,176/4,270	**1.21 (1.08–1.37)**	859/4,748	1.09 (0.99–1.20)	859/4,748	0.90 (0.81–1.00)	859/4,748	1.07 (0.97–1.19)
Diabetes	89/5,935	0.85 (0.63–1.15)	70/5,935	0.98 (0.67–1.43)	89/5,935	1.21 (0.79–1.86)	70/5,935	0.76 (0.50–1.16)	70/5,935	1.16 (0.77–1.77)	70/5,935	1.12 (0.76–1.63)
Major depressive disorder	400/4,649	1.00 (0.88–1.13)	292/5,146	1.07 (0.88–1.33)	400/4,649	1.17 (0.96–1.42)	292/5,146	0.95 (0.80–1.14)	292/5,146	1.11 (0.93–1.33)	292/5,146	0.87 (0.73–1.04)
Generalized anxiety disorder	555/4,704	1.01 (0.91–1.13)	394/5,208	1.09 (0.92–1.30)	555/4,704	1.08 (0.92–1.27)	394/5,208	**0.80** (**0.69–0.92)**	394/5,208	**1.31 (1.13–1.52)**	394/5,208	**0.84 (0.72–0.98)**

^a^*N* differs by sleep measure because not all Fitbit devices report restless sleep or sleep stages and because participants with ≤15 days of monitoring in the ‘baseline’ period were excluded from the models for average sleep duration and sleep irregularity but included in the restless sleep duration and sleep stages models to increase the sample size.

^b^HR (95% CI) values in bold indicate significant associations. All Cox proportional hazards models were adjusted for age, sex, baseline BMI, baseline systolic blood pressure, smoking status, alcohol drinking status, education status, time-varying average daily step count and previous diagnoses of cancer or coronary artery disease.

**Extended Data Fig. 4:**
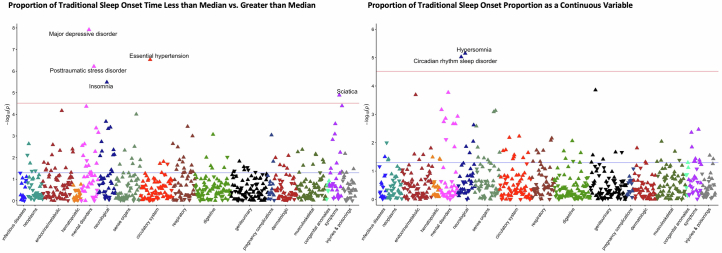
Phenome-wide analyses exploring relationship between traditional sleep onset times (8:00 PM to 2:00 AM) and incident disease. All phenome-wide analyses were performed using multiple logistic regression models adjusted for age, sex, average daily step count across the entire monitoring period, and EHR length. All reported P-values were based on two-tailed probability. Bonferroni significance line of 3.06 × 10–5 is indicated by red line and P-value of 0.05 is indicated by the blue line. Upwards triangles indicate odds ratios > 1 and downwards triangles indicated odds ratios < 1.

In stratified analyses of the significant associations (Extended Data Fig. 4), we observed that sleep duration and sleep irregularity were most associated with risk for obesity in participants who were younger than 50 years, male, white or lived in the Northeast (versus West, South, Midwest). Increased Fitbit-derived deep or light sleep percentages were most associated with risk of generalized anxiety disorder in participants who were older than 50 years, female, white or lived in the Northeast. Increased REM sleep percentage was associated with reduced risk of generalized anxiety disorder in participants who were younger than 50 years, female, white and lived in the Northeast. Stratified analyses for associations between Fitbit-derived sleep stages and incident atrial fibrillation or heart failure showed no major differences, albeit several of the stratum had fewer than 20 cases and could not be reported because of AoU data and statistics dissemination policies.

**Fig. 4:**
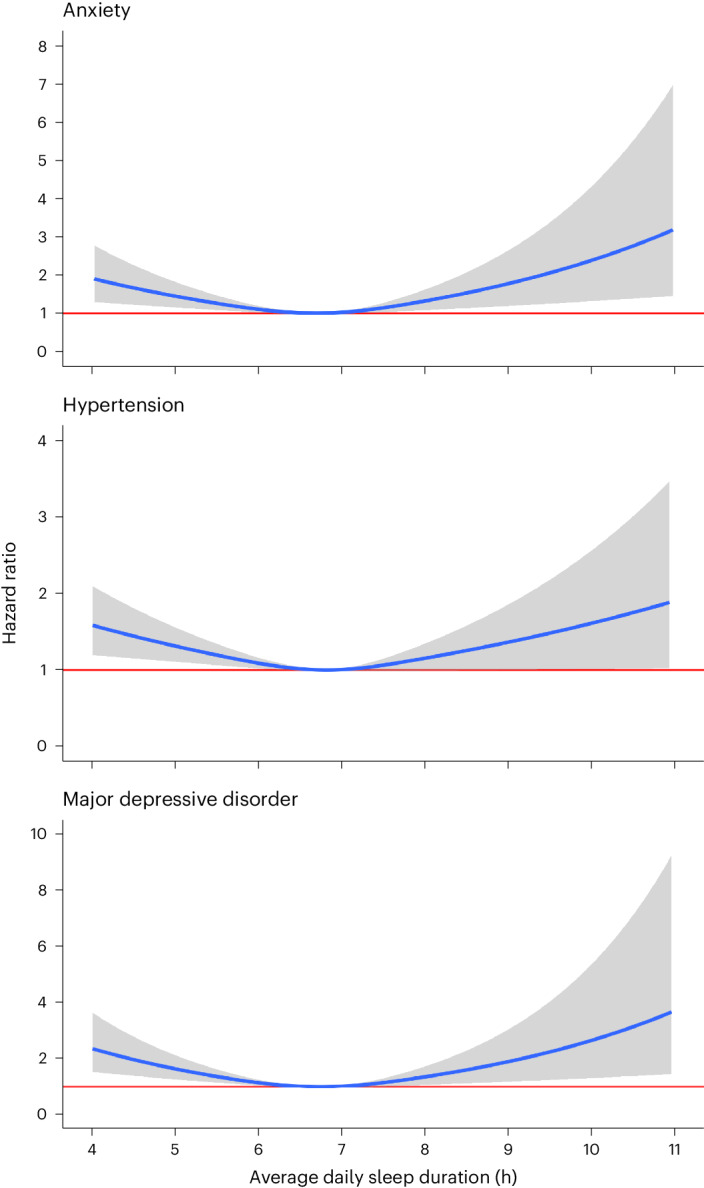
J-shaped relationship between average daily sleep duration and chronic diseases. Cox proportional hazard models were used to compute HR values as a function of average daily sleep duration and plotted as blue curves for generalized anxiety disorder, hypertension and major depressive disorder. The median of average daily sleep (6.8 h) was used as the reference. Gray area indicates 95% CI. The red horizontal line indicates an HR of 1. All Cox proportional hazards models were adjusted for age, sex, baseline BMI, baseline systolic blood pressure, smoking status, alcohol drinking status, education status, time-varying average daily step count and previous diagnoses of cancer or coronary artery disease.

We identified significant nonlinear, J-shaped relationships between average daily sleep duration and hypertension (*P* for nonlinearity = 0.003), major depressive disorder (*P* < 0.001) and generalized anxiety disorder (*P* < 0.001) (Fig. 4). Compared with the median of average daily sleep (6.8 h), participants who had an average daily sleep duration of 5 h had 29% increased risk of hypertension (HR = 1.29; 95% CI = 1.09–1.54), 64% increased risk of major depressive disorder (1.64; 1.27–2.12) and 46% increased risk of generalized anxiety disorder (1.46; 1.16–1.83) (Extended Data Table 2). Participants with an average daily sleep duration of 10 h had 61% increased risk of hypertension (1.61; 1.01–2.58), 163% increased risk of major depressive disorder (2.63; 1.31–5.31) and 130% increased risk of generalized anxiety disorder (2.30; 1.27–4.17).

### Sensitivity analysis for sleep apnea

Previous studies have shown that obstructive sleep apnea is highly associated with atrial fibrillation and several cardiometabolic disorders, including obesity, diabetes and hypertension^29,30^. To assess the influence of sleep apnea in our findings, we showed that a majority (27 of 41) of reported PheWAS associations were still significant after adjustment for previous diagnoses of sleep apnea (Extended Data Table 3). Increased REM sleep percentage remained associated with lower odds of atrial fibrillation and increased sleep irregularity remained associated with increased odds of several psychiatric (major depressive disorder, bipolar disorder, generalized anxiety disorder) and cardiometabolic disorders (obesity, essential hypertension). There remained an inverse association between deep sleep percentage and atrial fibrillation (OR = 0.86; 95% CI = 0.81–0.93; *P* = 3.98 × 10^−5^), but the association did not reach the Bonferroni significance threshold of 3.06 × 10^−5^.

After adjusting the Cox proportional hazard models for previous diagnosis of sleep apnea, a majority (8 of 9) of the reported associations remained significant (Extended Data Table 4). In addition, three of the Fitbit-derived sleep metrics were now significantly associated with obesity after adjustment for sleep apnea, including average restless sleep duration (HR = 1.14; CI = 1.01–1.29), average REM sleep percentage (1.12; 1.01–1.23) and average light sleep percentage (0.88; 0.79–0.97). The inverse association between deep sleep percentage and atrial fibrillation remained significant (0.59; 0.35–0.99).

### Sensitivity analysis for atrial fibrillation

It is possible that the atrial fibrillation associations we observed were an artifact of the Fitbit algorithm, which assigns sleep stages based, in part, on heart rate. The Fitbit devices used by AoU participants do not report heart rate irregularity. Therefore, to examine the potential for atrial fibrillation during sleep, we calculated and compared heart rate variability (standard deviation of average normal-to-normal intervals (SDANN)) among participants with and without atrial fibrillation during sleep and wake times. Higher heart rate variability may suggest a higher preponderance of atrial fibrillation during sleep. There were no significant differences in heart rate or heart rate variability during sleep or wake times between participants with atrial fibrillation and matched controls without atrial fibrillation. However, we did observe lower SDANN during REM (median 3.6 versus 3.8; *P* = 0.021) and deep sleep (3.1 versus 3.2; *P* = 0.019) among those with atrial fibrillation (Extended Data Table 5). In addition, participants with atrial fibrillation were more likely to have prevalent cardiometabolic disease, including coronary artery disease (30% versus 11%; *P* < 0.001) and obesity (39% versus 30%; *P* = 0.014).

## Discussion

We conducted the largest study to date that analyzes sleep patterns objectively and longitudinally across many years using direct measures of sleep from commercial wearable devices linked to EHR data. We observed clinically and statistically significant relationships between sleep quantity, quality and regularity and the onset of important chronic diseases even after accounting for daily activity (that is, step counts).

Our discovery analyses demonstrated several expected findings, such as the association between increased restless sleep duration and increased odds of incident sleep disorders or insomnia. Similarly, participants who had fewer sleep onset times between 8:00 p.m. and 2:00 a.m. had higher odds of incident sleep disorders, including insomnia, hypersomnia and circadian rhythm disorders. In addition, many of our findings are supported by previous studies using large-scale population surveys, such as our observations that decreased daily sleep duration and increased sleep irregularity are associated with obesity and sleep apnea^4,7^. Daily average sleep duration had nonlinear, J-shaped associations with hypertension, major depressive disorder and generalized anxiety disorder, a well-documented phenomenon reported in previous studies^1–6,31,32^. Our findings, along with those from previous epidemiologic studies, support the notion that 7 h of objectively measured sleep may be the middle of the healthy range for adults rather than the floor^33^, highlighting that the range of healthy sleep depends on the tool used to measure sleep.

Sleep irregularity has been difficult to study because of the limitations of data derived from cross-sectional surveys or polysomnograms and the infeasibility of obtaining objective measurements in the general population before the availability of modern consumer wearables. A 2023 study showed that sleep irregularity was associated with all-cause mortality in the UK Biobank, a large biobank linked to EHRs in the United Kingdom^8^. However, in that study, sleep patterns were only monitored for a 7-day period and were derived from wearable actigraphy data, which is less accurate than Fitbit objective measures of sleep^8,17–19,21^. Another study demonstrated associations between sleep irregularity and hypertension using data collected across 9 months with under-the-bed monitors^31^. Our study showed similar findings using a larger sample of person-nights and identified many additional associations across the phenome that are consistent with previous cross-sectional studies^12,25,34,35^. Furthermore, we showed that a lower proportion of ‘traditional’ sleep onset times was associated with hypertension and several psychiatric disorders. Notably, there were more associations for sleep irregularity than any other sleep pattern included in our study. One possible explanation could be that sleep irregularity can contribute to desynchronization of sleep–wake cycles and circadian rhythms^36^. Circadian rhythm disruptions are linked to a number of adverse health outcomes and have negative effects on human physiology, such as reduced insulin sensitivity, increased inflammation and downregulation of serotonin receptors^37^. We also observed that the majority of associations related to sleep irregularity were still significant after adjusting for average daily sleep duration, suggesting that sleep irregularity is an independent risk factor and highlighting the importance of longitudinal sleep monitoring for healthy sleep patterns.

There is conflicting evidence regarding the connection between REM or deep sleep and atrial fibrillation. In the Multi-Ethnic Study of Atherosclerosis cohort (*N* = 2,048), longer polysomnographic slow-wave (deep) sleep was associated with decreased atrial fibrillation prevalence but REM sleep was not^38^. By contrast, the Health eHeart study (*N* = 1,127) found that longer REM sleep was associated with decreased atrial fibrillation prevalence but longer deep sleep was not^39^. As the largest of these studies and the only one with longitudinal data, we found that increased Fitbit-derived REM and deep sleep duration were both associated with decreased risk of incident atrial fibrillation in our discovery analyses but only the deep sleep association was significant in time-varying analyses. Sleep stages may be linked to atrial fibrillation via their influence on signaling from the autonomic nervous system to the heart. The sympathetic and parasympathetic nervous systems are both involved in the development of atrial fibrillation^40^. Sympathetic activity is high during REM sleep, whereas parasympathetic activity is high during deep sleep^41^. It is possible that decreased REM or deep sleep may lead to abnormal autonomic nervous system signaling to the heart, increasing the risk of atrial fibrillation. However, it is important to acknowledge that Fitbit identifies sleep stages by monitoring heart rate and movement, which may be affected in participants with atrial fibrillation or cardiac conduction and cardiovascular abnormalities. In our study, there was a higher prevalence of coronary artery disease and obesity in participants with atrial fibrillation, even after matching for age, sex and baseline body mass index (BMI). Our findings that decreased proportions of REM or deep sleep are associated with incident atrial fibrillation may instead be identifying markers for occult atrial fibrillation and/or poor overall cardiovascular health. More longitudinal studies are needed to elucidate the relationship between Fitbit-derived sleep stages and atrial fibrillation. If confirmed, these findings have the potential to inform clinical guidance on sleep hygiene to reduce atrial fibrillation incidence or recurrence in participants at high risk.

Previous studies have shown that obstructive sleep apnea is highly prevalent among patients with atrial fibrillation and several cardiometabolic disorders, including obesity, diabetes and hypertension^29,30^. We observed that the majority of our reported findings for Fitbit-derived sleep metrics remained significant after adjusting for previous diagnosis of sleep apnea in both the discovery-focused PheWAS and time-varying analyses, suggesting that sleep patterns are risk factors for chronic disease independent of sleep apnea.

There are several limitations to this study. First, the study cohort is relatively young, majority female, white and college educated. The generalizability of the findings to underrepresented communities or those in areas of deprivation is unclear and, thus, a high priority for future studies. Notably, there is an active effort within the AoU Research Program to expand the diversity of participants with Fitbit data through the WEAR study by providing free Fitbit devices to invited participants from underrepresented communities. Nonetheless, many of our findings are supported by previous studies using diverse populations with survey or polysomnogram data. In addition, our findings will be highly relevant to the increasing proportion of the general US population that owns a commercial wearable device, which reached nearly 30% in 2020^42^. Moreover, because the owners of commercial wearable devices are broadly healthier than the general population, the reported effect sizes and impact of poor sleep health in this study may actually be stronger in the general population^22^. Second, the sleep data included in our analyses are reported from and calculated by Fitbit. The Fitbit algorithms have been evaluated against gold-standard polysomnograms in many studies^14–20,43,44^. The largest of these validation studies showed that Fitbit did not significantly differ in estimation of total sleep time or deep sleep compared with polysomnograms, but underestimated REM sleep by 11.4 min (ref. ^18^). Therefore, our findings may not generalize to sleep data from non-Fitbit sources because of potential systematic misestimation of sleep stage proportions^14–20,43,44^. Although there may be inherent biases in Fitbit’s estimation of sleep stages compared with polysomnograms, the Fitbit algorithm has not been changed since launching in 2017. Therefore, such biases would likely manifest in the same way in the same individual over time. Nonetheless, our findings are consistent with those from studies using polysomnograms, highlighting that Fitbit devices still meaningfully capture the fundamental aspects of sleep as it relates to health and disease despite modest reductions in performance compared with polysomnograms. It is also important to consider that polysomnogram-assigned sleep stages are limited by high inter- and intra-rater variability^45,46^. Third, we cannot exclude the possibility that our findings are the result of reverse causation. We focused on incident diseases after the first 180 days of the sleep monitoring period to mitigate this risk. It is also possible that sleep disturbances may be an early indicator for some conditions identified in the analysis, such as obstructive sleep apnea, gastroesophageal reflux disease or asthma. Fourth, there were some associations identified in the PheWAS that did not achieve significance in the time-varying analyses. This may be due to various aspects including the characteristics of the statistical model, available covariates and the population sample analyzed. Fifth, we acknowledge the limitations of using diagnosis codes from EHRs as outcomes and the possibility of misclassification. Lastly, there are other potential confounders that we are unable to account for because of the nature of the AoU data resource and individual patterns of commercial wearable devices usage, such as the variability in participants’ occupations.

Despite these limitations, the unique features of our study design and data sources set it apart from previous studies, yielding results that are both novel and clinically relevant. To our knowledge, this is the largest study to objectively analyze sleep patterns longitudinally across many years using direct measures of sleep. Using data from commercial wearable devices, we were able to assess detailed sleep patterns across nearly 6.5 million person-nights, which was previously not possible with surveys or was prohibitively expensive with polysomnograms. Previous studies have often focused on a narrow set of phenotypes, whereas we are able to analyze associations across 1,636 diverse phenotypes because of the AoU’s linkage of sleep data with EHR data. This study also includes time-varying analyses, which account for changes in sleep behavior over time, unlike previous cross-sectional studies. Finally, our study also accounts for the impact of sleep on disease risk accounting for concomitant, longitudinal activity behavior, which is novel compared with other studies and shows the independent impact of sleep.

Our study helps advance the current understanding on the relationship between behavior and health and has important clinical and public health implications. Wearable devices with sleep monitoring capabilities are becoming increasingly popular. Our results, which are based on sleep patterns directly reported to consumers, will be highly relevant for participants monitoring their sleep and for providers counseling on healthy sleep habits. The integration of patient-generated sleep data with EHRs in routine care in the future could enable providers to monitor changes in sleep patterns as early disease indicators and to provide evidence-based guidance tailored to an individual’s unique clinical circumstances and risk profile.

In summary, we show that insufficient sleep quantity, quality and regularity are all associated with increased incidence of numerous chronic diseases, including obesity, atrial fibrillation, hypertension, major depressive disorder and generalized anxiety disorder. These findings, if validated, may provide evidence for updated recommendations on healthy sleeping habits, especially in individuals at high risk for chronic conditions. Furthermore, our study supports the value of integrating data from commercial wearable device with EHRs to advance scientific discoveries and to improve patient care.

## Methods

### Study participants

All study participants consented to participate in the All of Us Research Program, which has been described at length elsewhere^26,27^. Briefly, all adults (≥18 years) in the United States are eligible to enroll in the AoU research programs, except for individuals who are in prison or are unable to consent on their own. For this study, we used registered and controlled tier data (C2022Q4R9) available on the AoU Researcher Workbench. Controlled tier data were used only for analyses that required absolute dates. Data from 413,457 individuals who were enrolled from May 2018 to July 2022 were available at the time of analysis. Detailed information was collected, including participant demographics and survey data during the digital enrollment, physical measurements and vital signs measured at a partnered healthcare provider organization, voluntarily shared EHR data from partnered healthcare provider organization, and voluntarily shared Fitbit device data by linking their Google Fitbit account in the AoU participant portal^26,27,47^.

### Fitbit sleep data

We used the Fitbit-derived daily summary data that are displayed to consumers, including daily duration of sleep time, restless sleep time (defined by Fitbit as sleep with movement but not indicating wakefulness) and Fitbit-derived sleep stages (light, deep, REM). Fitbit estimates sleep stages using a proprietary algorithm based on heart rate and movement and only estimates sleep stages for sleep periods of duration >3 h (ref. ^43^). Based on algorithm validation studies, Fitbit maps ‘light’ sleep to N1 + N2, ‘deep’ sleep to N3, and ‘REM’ to rapid eye movement sleep^43^.

For the discovery analyses, we averaged daily sleep patterns for each participant across their entire monitoring period. Only sleep periods with available Fitbit-derived sleep stage data were used to estimate average daily sleep stages. The Fitbit monitoring period began when the participant created a Fitbit account, not on the date of enrollment in AoU. Therefore, the initiation of monitoring precedes AoU enrollment in participants. We defined sleep irregularity as the s.d. of the daily sleep duration. We calculated a daily percentage (time in sleep stage/total sleep time) for each sleep stage. To characterize typical sleep schedule patterns, we calculated the proportion of sleep onset during ‘traditional’ times, defined as between 8:00 p.m. and 2:00 a.m., which was chosen because the median sleep onset in our cohort was 11:10 p.m. and a large proportion of sleep onset times were within this timeframe when plotted on a heatmap (Fig. 1); weekends were excluded from the analysis of ‘traditional’ sleep onset times because of their low representation of typical sleep schedules.

### Quality control

We included adult (≥18 years) participants with EHR data and at least six months of Fitbit sleep data from the start to the end of each participant’s Fitbit monitoring period. To enrich for consistent device wearers and ensure our data provided an accurate reflection of typical and realistic sleep patterns, we excluded participants who had <4 h of sleep data on ≥30% of days. These thresholds were informed by mathematical models as the theoretical cutoff at which insufficient sleep would be unsustainable^48^. We only included sleep periods that were flagged as the ‘main sleep’ (that is, longest sleep per day) by the Fitbit algorithm. We also only included data from dates for which both step and sleep data were available to align both variables for time-varying analyses.

### Study outcomes and covariates

The primary outcomes were incident diagnoses coded in the EHR. We excluded incident diagnoses in the first 180 days of Fitbit monitoring to reduce the risk of reverse causation, assuming those conditions already existed but were not yet reflected in billing codes. Incident diagnoses were identified by International Classification of Diseases billing codes and mapped to 1,636 phecodes^49–51^. Phecodes consolidate similar International Classification of Diseases codes to reduce collinearity and multiple comparisons. Nonspecific phecodes (for example, 512.9 = Other dyspnea) were not reported. Phecodes with ≤20 cases were not reported to comply with AoU Data and Statistics dissemination policies.

Participant demographics (age, self-reported sex, self-reported race/ethnicity, education) and lifestyle factors (smoking, alcohol intake) were derived from survey data completed at the time of enrollment. Average daily step count was derived from the Fitbit physical activity data, as previously described^22^. We calculated total EHR length as the time between the first and last billing code (diagnosis or procedure), vital sign documentation or laboratory measurement in the participant’s EHR.

### Statistical analyses

Discovery-focused PheWAS were performed for each sleep pattern averaged across the entire Fitbit monitoring period using multiple logistic regression models. For the analysis of a given disease of interest, we excluded participants with any previous diagnosis of that particular disease of interest before Fitbit monitoring or any incident diagnoses of that particular disease of interest within the initial 180 days of Fitbit monitoring to mitigate the risk for reverse causation. After Bonferroni correction for multiple comparisons, the significance threshold was α < 0.05/1,636 = 3.06 × 10^−5^. PheWAS analyses were adjusted for age, sex, average daily step count across the entire monitoring period and EHR length.

We performed Cox proportional hazards regression models for incident phecodes by sleep pattern for chronic disease phenotypes of interest, selected a priori from reported associations in previous studies^1–7,28^. We excluded participants with any previous diagnosis of the chronic disease phenotype before Fitbit monitoring or any incident diagnoses within the initial 180 days of Fitbit monitoring to mitigate the risk for reverse causation. HR values and 95% CI were calculated comparing the 75th and 25th percentile of each sleep pattern. Participants were censored at their last medical encounter, which was defined as their last billing code, recorded vital sign or laboratory measurement. We established the ‘baseline’ sleep patterns and steps counts as the average across the initial 180 days of Fitbit monitoring for each individual. Participants with ≤15 days of monitoring in the ‘baseline’ period were excluded from the models for average sleep duration and sleep irregularity, but those individuals were included in the restless sleep duration and sleep stages models to increase the sample size because not all Fitbit devices report restless sleep duration or sleep stages. Sleep patterns and step counts were averaged on a monthly basis starting from the last date of the 180-day ‘baseline’ period and extending to the date of censor/incident diagnosis. The monthly averages were entered into the Cox models as time-varying variables. Months with ≤15 days of observations were excluded. Wald *χ*^2^ tests were used to assess for nonlinear relationships between sleep patterns and chronic diseases. We calculated HRs as a function of each sleep metric when compared with the median of that sleep metric in the filtered cohort. All Cox models were adjusted for age, sex, baseline BMI, baseline systolic blood pressure, smoking status, alcohol drinking status, education status, time-varying average daily step count and previous diagnoses of cancer or coronary artery disease. Missing data for covariates were imputed using multiple imputation with predictive mean matching. Continuous variables were modeled as restricted cubic splines with 3-knots.

Statistical analyses were performed in R (v.4.2.2, R Project https://www.r-project.org) on the AoU Researcher Workbench, a secure cloud-based platform. Statistical tests were based on two-tailed probability.

### Sleep apnea sensitivity analyses

Sensitivity analyses were performed to assess for the influence of sleep apnea on our findings. Discovery PheWAS and Cox proportional hazard regression models were repeated for each Fitbit-derived sleep metric as described above with an additional covariate for previous diagnosis of sleep apnea.

### Sleep stage-specific sensitivity analyses

Analyses showing an association between sleep stage and atrial fibrillation were subjected to a sensitivity analysis because of concern for either active atrial fibrillation or algorithm-based misclassification driving the association^43^. We compared heart rate variability (SDANN) between participants with previous diagnosis of atrial fibrillation (excluding those with pacemakers) and matched controls without previous diagnosis of atrial fibrillation. Average minute-level heart rate was computed for consecutive 5-min intervals over a 24-h period (00:00 to 23:59). The average RR interval duration was inferred by dividing each average heart rate by 1/6,000. The SDANN was then estimated by taking the s.d. across all average RR interval durations for the entire day^52^. Controls were matched on age, sex and nearest BMI measurement to baseline using Mahalanobis distance matching.

### Reporting summary

Further information on research design is available in the Nature Portfolio Reporting Summary linked to this article.

## Online content

Any methods, additional references, Nature Portfolio reporting summaries, source data, extended data, supplementary information, acknowledgements, peer review information; details of author contributions and competing interests; and statements of data and code availability are available at 10.1038/s41591-024-03155-8.

## Supplementary information

Reporting Summary

## Figures and Tables

**Extended Data Table 1:**
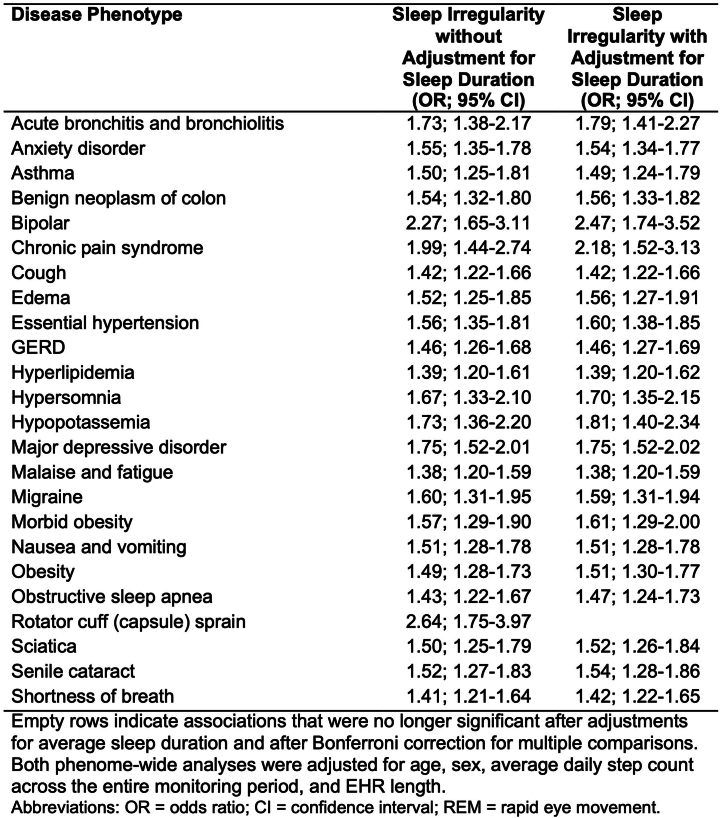
Table of significant associations between sleep irregularity and disease phenotypes with and without adjustment for average daily sleep duration

**Extended Data Table 2:**
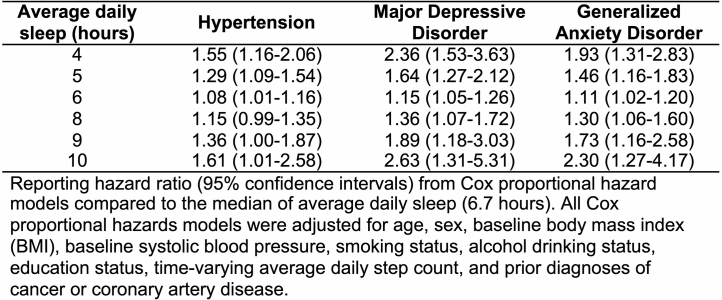
Table of hazard ratios at different average daily sleep thresholds for hypertension, major depressive disorder, and generalized anxiety disorder

**Extended Data Table 3:**
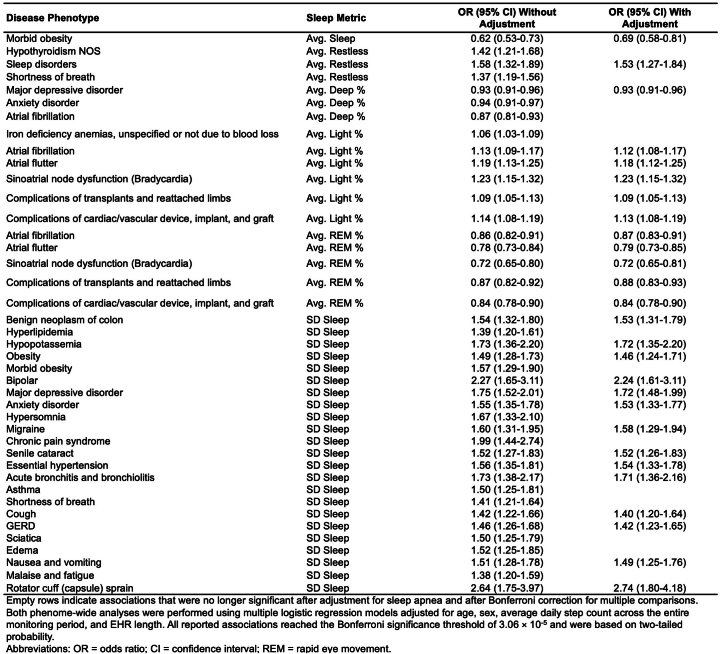
Table of significant associations between Fitbit-derived sleep metrics and disease phenotypes from discovery-focused phenome-wide association analyses with and without adjustment for sleep apnea

**Extended Data Table 4:**
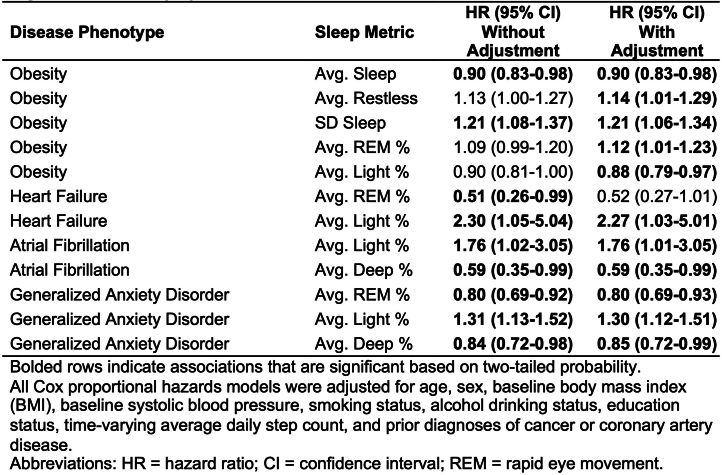
Table of significant associations between Fitbit-derived sleep metrics and disease phenotypes from Cox proportional-hazard models with and without adjustment for sleep apnea

**Extended Data Table 5:**
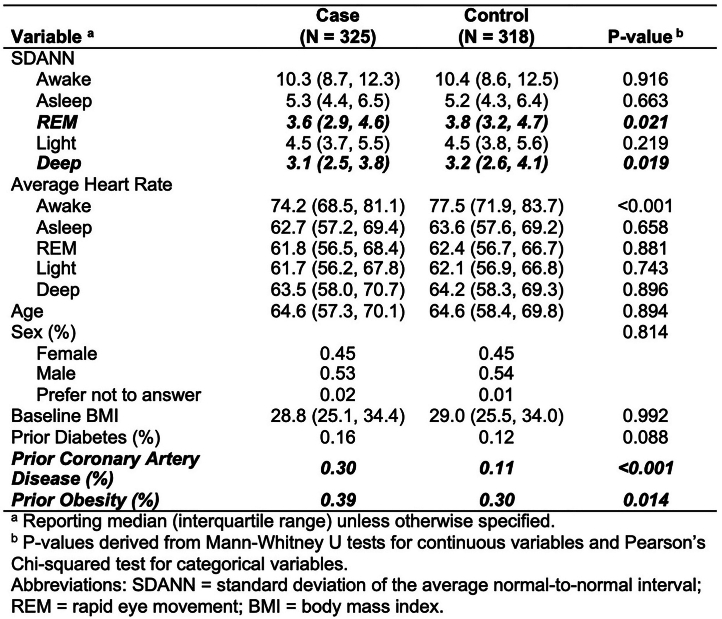
Table of heart rate variability (standard deviation of average normal-to-normal interval [SDANN]) for patients with atrial fibrillation and matched controls

## Data Availability

To ensure privacy of participants, data used for this study are available to approved researchers following registration, completion of ethics training and attestation of a data use agreement through the All of Us Research Workbench platform, which can be accessed via https://workbench.researchallofus.org/.

## Code availability

Code used for this study can be made immediately available to any approved researchers on the All of Us Research Workbench platform by contacting our study team.
